# Hygienic and Prophylactic Medicine

**Published:** 1874-07

**Authors:** R. J. Preston

**Affiliations:** Virginia


Vol. IV.—JULY, 1874.—No. 7.

### Original Communications.

### "HYGIENIC AND PROPHYLACTIC MEDICINE."

BY R. J. PRESTON, M.D., OF VIRGINIA.

*Read before the Abingdon Academy of Medicine, April 6th, 1874.*

*Mr. President and Fellows of the Abingdon Academy of Medicine:*

Gentlemen,—The subject which you have selected for discussion this evening, and which I have also chosen as the subject of my essay before you is "Hygienic and Prophylactic Medicine." There is here a seeming contradiction of terms, as the popular mind naturally excludes medicines from its idea of health; but properly speaking this is the grandest department in the science of medicine, and we of the profession feel an exultant pride as we look back over the history and records of the last half century and see that it is here that our greatest energies and talents have been put forth, and our most glorious triumphs have been achieved. Hygiene proper, relates to the preservation of health; Prophylaxis to the prevention of disease. But hygiene alone in a wider sense embraces almost the whole domain of medicine, and "is divided into three parts (1) *Prophylacticine,* which forsees and prevents diseases; *Synteritice* employed in preserving health; and *Analaptice* whose office is to cure diseases and restore health."

It is our purpose however in the consideration of the subject to-night to confine ourselves to the general acceptation of these terms, viz: the preservation of health, and the prevention of disease. We shall endeavor to point out in as brief a manner as possible some of the advances which have been made in this department of medicine, their great practical utility to mankind—and the still greater good and utility yet attainable, could the popular mind be educated and brought up to a proper appreciation of those laws of hygiene which regulate and control their own well-being, and to the practical and universal application of them in every-day life. To show the beneficial results accruing to mankind from advances in any department of medicine, we must necessarily consider in most instances, the aggregate results from all causes, and then eliminate and assign to each its due proportion of the whole.

From English statistics it is shown that "toward the middle of the last century, sixty out of every one hundred children born in London died before they reached the first year of age; but the mortality now is about thirty-five out of every one hundred for the same period. Out of six hundred thousand children born annually in Great Britain, three hundred thousand would have perished; now about two hundred thousand die; thus showing a saving of one hundred thousand human lives a year in Great Britain. In Geneva records have been kept since 1590, and it has been ascertained that a child has now five times greater chance of living to the age of twenty-one, than it had in the sixteenth century. Out of six hundred thousand accouchments yearly in Great Britain only about three thousaud mothers now perish; whereas by the mortality rate of the last century eleven thousand or twelve thousand maternal lives would be lost. This saving of life is almost entirely due to improved hygienic and prophylactic measures, and "we may proudly point to this modern advancement in medical science, affecting as it does in this last item alone, a saving of eight or nine thousand mothers yearly in Great Britain. The mortality in the army years ago was immense—since the Crimean war the mortality in the English guards has fallen from twenty per cent. to nine per cent., and in the infantry from eighteen per cent. to eight per cent. The statistics of London show that by sanitary measures chiefly the mortality in some of the parishes has been reduced from five per cent. to one and a half per cent. (R. & S. Med. Jour.)"

Not to weary with too numerous citations there is one other advancement in prophylactic medicine to which the physician may proudly point as the greatest discovery of any age—the discovery of vaccination by Jenner in 1798. "In the history of medicine before this discovery, we are told that small-pox was a terror to mankind, sweeping over the land like fire over the prairies, smiting down prince and peasant. About 1519 in Mexico three and a half millions of the population were suddenly carried off; in quito alone it destroyed one hundred thousand Indians in 1563; in Iceland in 1707, eighteen hundred perished out of fifty thousand population, and in Europe alone the annual mortality amounted to half a million. One-third of those attacked died, and it destroyed, maimed and disfigured one-fourth of mankind."

Such is the immunity given from this terrible scourge by this discovery, that now it is no longer feared. Indeed so perfect has been the security that mankind have fallen into a forgetfulness and disregard of this great prophylactic measure, until they now and then have been startled, and awakened to its great importance and the great danger of disregarding it. It has been said that "the lancet of Jenner, armed with the cow-pox matter has saved in the world more human lives, than gunpowder and the sword ever destroyed during any century in the history of mankind!" Then let us say as a passing tribute to his memory, all honor to the immortal Jenner—illustrious benefactor of his race, and most worthy disciple of the Esculapian art.

These are some of the results of the recent advances in medicine, and many others equally striking and wonderful might be enumerated had we the time. It has been said that "the advances have been most astonishing in every department of medicine except therapeutics, and that it carries but little comfort to the popular mind to be told that a great deal more is known about disease, except in the matter of curing it than used to be known." All these advances however have been such as to contribute most powerfully and directly to the well-being of mankind, and there have been many improvements too in therapeutics, and much more is known even in the matter of curing disease. It is true that in the light of recent discoveries therapeutics dwindles to a subordinate place in medical science, and the physician to-day relies more often upon hygiene and phrophylaxis in prosecuting his calling, than he does upon the articles of his materia medica. The old adage that "an ounce of prevention is better than a pound of cure" has become a scientific maxim, and every physician and every intelligent person recognises the fact "that prevention is much easier and much more feasible than cure." The well-being of an individual is not a mystery, shrouded in abstruse technicalities which a physician only can solve or comprehend, "but it is a most practical matter which rests mainly with himself, and the conditions of which it behooves him to well understand." This brings us in the consideration of this subject to the practical question how to preserve health and prevent disease. The chief essentials for this, consists in pure air, pure water, proper food and proper clothing. In supplying these essentials, as nearly as can be done, the two latter rest mainly with the individual and must be left mainly to his discretion. But the two former rest with the community at large, and should concern every public officer and every intelligent person who has at heart his own well-being, and the well-being of the community in which he lives. By the code of medical ethics, which every true physician loves and reveres—we of the profession are the constituted guardians as it were of the public health, and are required "to be ever vigilant for the welfare of the community"—"to be ever ready to give counsel to the public in relation to medical police, public hygiene and legal medicine," and to enlighten the public in relation to drainage, ventilation, etc., etc., in regard to measures for the prevention of epidemic and contagious diseases."

"Every physician and every intelligent person recognises the fact, that to take care of their own health they must take care of the health of whole classes too ignorant or too weak to take care for themselves. The pestilence generated by the filth of illventilated and illdrained slums and sinks will poison the atmosphere of a whole community, and infect quarters at a considerable distance whose inhabitants may be properly observant of the conditions of health." To this extent then at least we are all our neighbors keeper, and "Carlyle's story of the poor woman whom rich men refused to recognize as a member of the human family and to succor, and who finally proved her sisterhood by infecting seven of them with typhus fever, has a sanitary as well as a moral lesson." From these facts we see the importance of strict sanitary regulations in towns and communities, regulating these things which are of vital importance to the health and well-being of all the inhabitants. A writer in the Journal of Chemistry says: "It is always possible to supply a city or town with good water, but in a village, where houses are few, money scarce and intelligence scarcer, it is a matter of exceeding difficulty, and will tax the energies of a health officer severely, for it is one of the hardest sanitary problems. The shallow wells and springs of villages are the pests of the country and many are as unsafe as loaded firearms. How often may one see in our rural districts the barnyard on the slope of a hill and the well or spring at the foot, where its waters are certain to catch all the foul filtrations from the manure heaps above?" And in most of our towns and villages how much worse is it than this? Not only barnyards and manure heaps, but privies, sinks and cesspools cover the whole face and sides of the hills, while the inhabitants use the water from the springs and wells beneath. "It is sickening when we come to observe and realize these things, and to think of the foul, sewerage-reeking soil through which the water percolates to its foul bed, and it is high time that a zealous and well organized crusade should be brought to bear upon them. The earth it is true is a good filter and disinfectant and will protect from contamination a long while, but it becomes saturated with decaying animal and vegetable matter, and no longer suited to perform this office, and the waters of wells and springs many yards distant may become impure and dangerous." It has been said by an experienced chemist that for one article of impure and dangerous food, a thousand samples of impure and dangerous water may be obtained in any part of the country. Prof. Husley says (Jour. Chem., May, 71) "that pestilences will only take their abode among those who have prepared unswept and ungarnished residences for them. London of 1665 and the cities of the east where the plague took up an enduring dwelling had narrow unwatered streets, with houses ill-drained, ill-lighted and ill-ventilated, while the inhabitants were ill-washed, ill-fed and ill-clothed. We in later times have learned somewhat of nature and partly obey her. Because of this natural knowledge and this fractional obedience we have no plague—because that knowledge is still very imperfect and that obedience yet more incomplete, typhus is our companion and cholera our visitor. It is not presumptious to express the belief that when our knowledge is more complete, and our obedience the full expression of that knowledge, typhus and cholera will disappear, as has the plague from among the inhabitants of London." To show how sadly incomplete is our obedience to laws now known, Capt. Galton has ascertained from the last census that the mortality in Great Britain "is one-third higher than it would be if our existing knowledge of the chief causes of disease were reasonably well applied throughout the country. There is a yearly average of one hundred and twenty thousand deaths in Great Britain from causes which may be termed preventible, and when we consider that the larger proportion of individuals attacked by these preventible diseases recover, and that only a small proportion die, it is oppressive to think of the enormous amount of human suffering and waste of human lives which these figures represent." "From a very careful analysis of the details of one thousand town families, and one thousand country families, Capt. Galton found that a town population supplies to the next generation only three-fourths the number of adults supplied by an equally numerous country population, and that in two generations the adult grand-children of a town population little more than half as numerous as those of a country population in a healthy district. Yet the sanitary conditions of these rural districts, 'he says,' is very far from perfect, and the majority of cottages which exist in rural parishes are deficient in almost every requisite that should constitute a home for a Christian family in a civilized community."—(Galaxy, Jan., 1874.)

From the recent investigations of Prof. Tyndall, on "dust and disease," we may learn concerning the nature of the impalpable powder which is hurled in our faces, and into our mouths on windy days. "The filthy excrementitious matter, associated with filaments of animal and vegetable waste, and minerals and mineral salts, which these investigations have revealed, constitute a compound neither healthy nor pleasant to inhale into the lungs or take into the stomach. And even the ordinary air we breathe, illuminated by the electric beam, appears as a semi-solid rather than a gas from the dust and matter suspended there. Nobody could, says Prof. Tyndall, in the first instance, without repugnance, place the mouth at the illuminated focus of the electric beam, and inhale the dirt revealed there. Nor is the disgust abolished by the reflection that although we do not see this nastiness, we are churning it in our lungs every hour and minute of our lives." 'Tis thus the germs of many contageous and epidemic diseases doubtless are received into the system. Prophylactic medicines comes to our rescue even here and provides the means by which we may filter the air of all these impurities, and by which so far as the germs are concerned, the air of the highest Alps may be brought into the chamber of the invalid. Prof. Tyndall has shown that the noxious air around patients in a crowded and infected district may be so filtered by cotton-wool respirators as to be restored to practical purity, and thus filtered, attendants may breathe it unharmed.

How far the practical results in this new field of prophylaxis may be extended remains to be seen, but the future of medicine in these departments, under the impetus given to our science by the increased energy of recent investigators is full of promise. "One difficulty after another will be surmounted in the years to come, and perhaps there will remain ulitmately but one great physical catastrophy which cannot be overcome by human skill, and that is death."

Then we of the medical profession everywhere should contribute our quota to the attainment of this end, and much may be done—widely-scattered as we are throughout every county and district—by keeping constantly before the public mind the known laws of hygiene and prophylaxis and strenuously insisting upon the intelligent application of them by towns, communities, cities and States. We will doubtless, as in every age, be traduced and slandered by the ignorant and uninformed, and have wrong motives imputed to us, but by faithful perseverance in this beneficent work we will accomplish much.

These things should be regulated and enforced by proper legislation and must be so done to be rendered thorougly effective. To this end we are encouraged by recent bills introduced in Congress looking to the establishment of a "National Bureau of Sanitary Science."

Although our own State is lagging behind her sister States in this respect, and is slow in seconding the efforts of our profession looking to this end; yet it is to be hoped that she, too, under the weight of argument impelling her and the welfare of her own citizens involved will soon come up to the full measure of the importance of this work. But while we hopefully await this action upon the part of our State, our efforts need not be abated or our hands idle. All these results are attainable by any town or community of themselves independent of State or general concert of action. They are attainable by us here in the town of Abingdon with but a small expenditure of means and money, blessed as we are by Providence with a climate unsurpassed, and a pure and invigorating mountain air. The last census of the United States show that south-west Virginia is one of the healthiest spots throughout this broad Union. But favored as we are in this respect, let us not say we need nothing more. Under the light of these discoveries can we here in Abingdon say that the water we drink is pure, receiving as it does the drainage and percolations of these "seven hills." Can we say that the air we breathe is pure, poisoned and contaminated as it is by the emanations from a hundred privies, manure heaps and cespools, reeking with decaying animal and vegetable matters? Only allow chemical analysis, and the light of the electric beam to make their revelations here, and I fear many loaded fire-arms would be found in our midst ready to destroy our lives only upon the application of the match. Then let us not mar the blessings we have from the hand of nature, but improve them and make (South-west Virginia), as it should be an Eldorado of health and happiness.

